# Synthesis and evaluation of the biostability and cell compatibility of novel conjugates of nucleobase, peptidic epitope, and saccharide

**DOI:** 10.3762/bjoc.11.145

**Published:** 2015-08-03

**Authors:** Dan Yuan, Xuewen Du, Junfeng Shi, Ning Zhou, Abdulgader Ahmed Baoum, Khalid Omar Al Footy, Khadija Omar Badahdah, Bing Xu

**Affiliations:** 1 Department of Chemistry, Brandeis University, 415 South Street, MS015, Waltham, MA 02453, USA; 2 Department of Chemistry, King Abdulaziz University, Jeddah, Saudi Arabia

**Keywords:** biocompatibility, biostability, nucleobase, peptidic epitope, saccharide

## Abstract

This article reports the synthesis of a new class of conjugates containing a nucleobase, a peptidic epitope, and a saccharide and the evalution of their gelation, biostability, and cell compatibility. We demonstrate a facile synthetic process, based on solid-phase peptide synthesis of nucleopeptides, to connect a saccharide with the nucleopeptides for producing the target conjugates. All the conjugates themselves (**1**–**8**) display excellent solubility in water without forming hydrogels. However, a mixture of **5** and **8** self-assembles to form nanofibers and results in a supramolecular hydrogel. The proteolytic stabilities of the conjugates depend on the functional peptidic epitopes. We found that TTPV is proteolytic resistant and LGFNI is susceptible to proteolysis. In addition, all the conjugates are compatible to the mammalian cells tested.

## Introduction

This article describes the synthesis and evaluation of a new class of molecular conjugates that consist of a nucleobase, a peptidic epitope, and a saccharide. Nucleobases, amino acids, and saccharides are part of the unified building blocks of life [1] because they constitute three key types of biomacromolecules–proteins, nucleic acids, and carbohydrates. Inspired by this molecular foundation resulted from evolution, we are developing biomaterials that consist of the covalent conjugates of these three classes of the basic building blocks of life. For example, we found that certain conjugates of nucleobase, amino acid, and saccharide (NAS) or some conjugates of nucleobase, saccharide, and amino acid (NSA) self-assemble in water to form supramolecular hydrogels [2–3], but, so far, none of the conjugates of saccharide, amino acid, and nucleobase (SAN) is able to act as hydrogelators [4]. Besides the properties of self-assembly, these conjugates are cell compatible [3–4]. Moreover, the NAS conjugates promote the proliferation of mES cells [5] and deliver the oligonucleotide into living cells [6]. Particularly, the incorporation of the functional peptidic epitope, RGD [4,7–8], results in a NAS conjugate that self-assembles in water, exhibits improved proteolytic stability [2], and promotes the development of mouse zygotes [5]. These results suggest that it is also worthwhile to incorporate other peptidic epitopes into the NAS conjugates and to evaluate their physiochemical and biological properties.

Based on the above rationale, we chose two short peptidic epitopes, TTPV and LGFNI, from two well-characterized proteins as the building blocks for nucleopeptides [9–13] or NAS conjugates. TTPV is from a calcium channel protein (stargazin) [14] and LGFNI is from a synapse associated protein 102 (SAP102) [15]. We connected these two functional peptide sequences with a nucleobase, and saccharide (or not). After investigating the gelation, biostability, and cell-compatibility properties of these conjugates (**1**–**8**), we found that all the conjugates exhibit excellent solubility in water without resulting in hydrogelation or precipitation. We observed that only the mixture of a proper pair of TTPV-containing (e.g., **5**) and LGFNI-containing (**8**) conjugates self-assembles to form nanofibers and results in a supramolecular hydrogel. Moreover, the conjugates containing TTPV or ETPV show excellent proteolytic stability, while the conjugates containing LGFNI undergo complete proteolysis catalyzed by proteinase K after 24 hours, with or without the presence of nucleobase or saccharide in the conjugates. Apparently, the stabilities of the conjugates coincide with their corresponding peptide sequences that TTPV is proteolytic stable and LGFNI proteolytic susceptible. In addition, all the compounds investigated in this work exhibit excellent compatibility to mammalian cells such as HeLa and PC12 cells. This work provides useful insights on the incorporation of peptidic epitopes into molecular conjugates that consist of a nucleobase, amino acids, and a saccharide for potentially developing new supramolecular biomaterials.

## Results and Discussion

### Molecular design

**Scheme 1:**
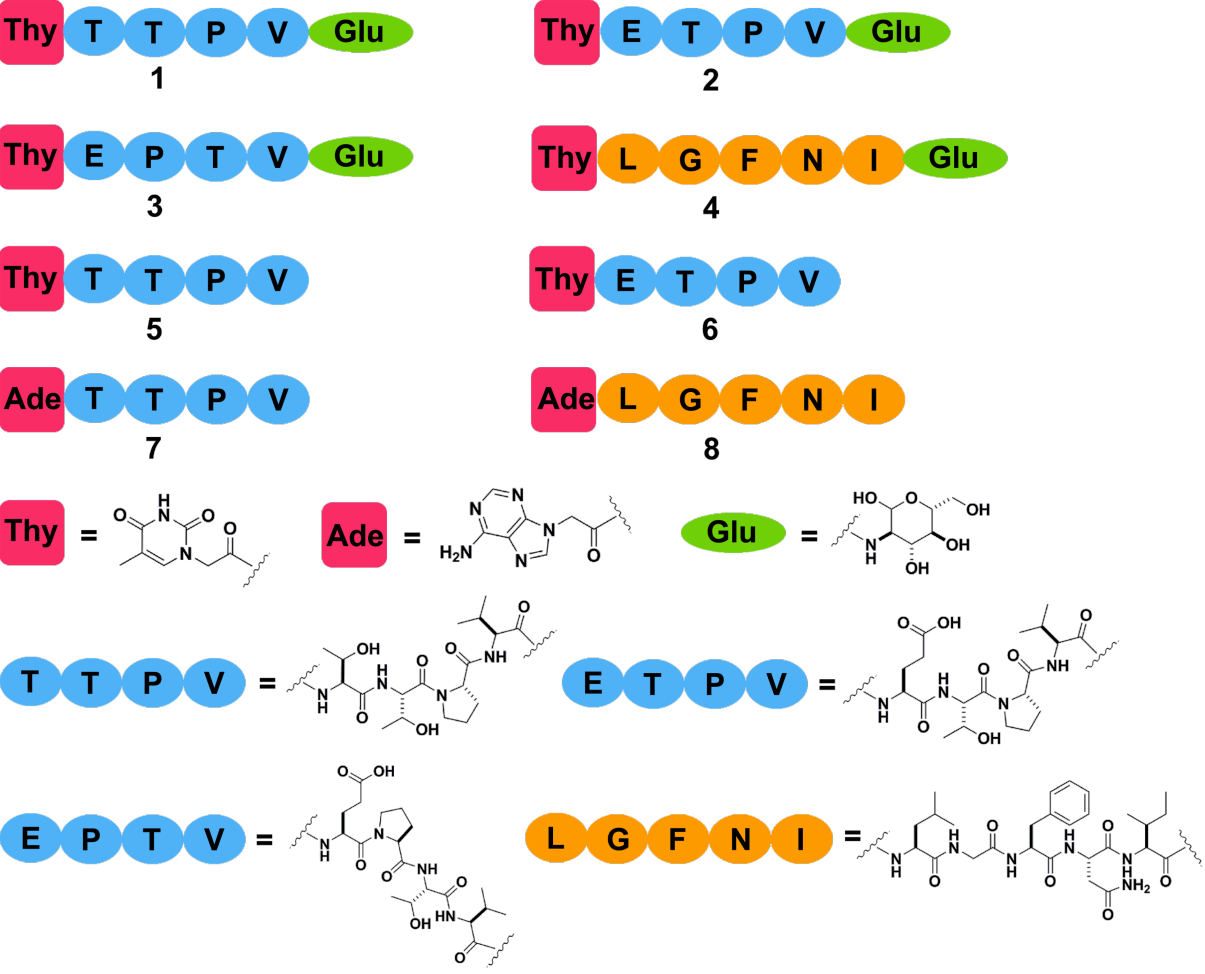
Chemical structures of the conjugates (nucleobase–amino acids–saccharide (NAS)), and nucleopeptides (NA).

Scheme 1 shows the chemical structures of the conjugates explored in this work. In the NAS conjugates **1**–**4**, we chose thymine as the nucleobase, TTPV and LGFNI, which are two well-characterized peptide binding motifs [16], as the peptidic epitopes, and glucosamine as the saccharide. As a control, we changed the sequence of peptides to ETPV or EPTV. To investigate the function of the saccharide, we designed the nucleopeptides **5** and **6**. In addition, we substituted thymine with adenine to generate nucleopeptides **7** and **8** that contain adenine, the nucleobase is complementary of thymine.

### Synthesis

**Scheme 2:**
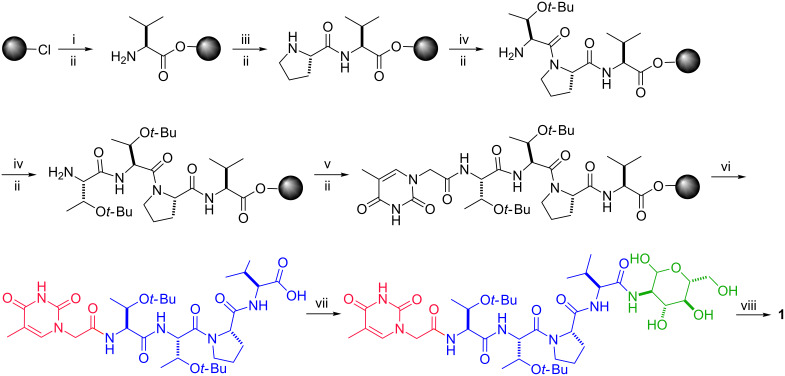
The representative synthesis route of conjugates NAS (**1**, including solid-phase peptide synthesis and liquid-phase synthesis). i) Fmoc-Val-OH, DIPEA; ii) 20% piperidine; iii) Fmoc-Pro-OH, HBTU, DIPEA; iv) Fmoc-Thr(*t*-Bu)-OH, HBTU, DIPEA; v) thymine-1-acetic acid, HBTU, DIPEA; vi) TFE/DCM 2:8; vii) D-glucosamine hydrochloride, HBTU, DIPEA; viii) TFA/H_2_O 95:5.

The NA conjugates **5**–**8** were obtained according to a facile method of solid-phase peptide synthesis (SPPS) [17]. The conjugates NAS were produced by a combination of SPPS and liquid phase synthesis. Scheme 2 shows a representative synthesis route of a NAS conjugate (**1**). We loaded the first amino acid, Fmoc-Val-OH, on 2-chlorotrityl chloride resin, then removed the Fmoc group with 20% piperidine in dimethylformamide (DMF) to expose the amino group. The second amino acid, Fmoc-Pro-OH, was reacted with the free amino group using the coupling reagent *N,N,N′,N′*-tetramethyl-*O-*(1*H*-benzotriazol-1-yl)uronium hexafluorophosphate/*N,N*-diisopropylethylamine (HBTU/DIPEA). The elongation of the peptide chain was done by repeating the removal of the Fmoc group and sequential addition of Fmoc-Thr(*t-*Bu)-OH, Fmoc-Thr(*t*-Bu)-OH and thymine-1-acetic acid. For the final step of the SPPS, we used 2,2,2-trifluoroethanol/dichloromethane (TFE/DCM 2:8) to cleave the fully protected NA from the resin. For conjugates **5**–**8**, we cleaved the chain from the resin with 95% trifluoroacetic acid (TFA) without N-protecting groups. Later, NAS was obtained by reacting D-glucosamine hydrochloride with the fully protected NA. After cleaving the protecting group on amino acids with 95% TFA, we used reversed-phase high-performance liquid chromatography (HPLC) to purify the target conjugates.

### Gelation properties

**Figure 1:**
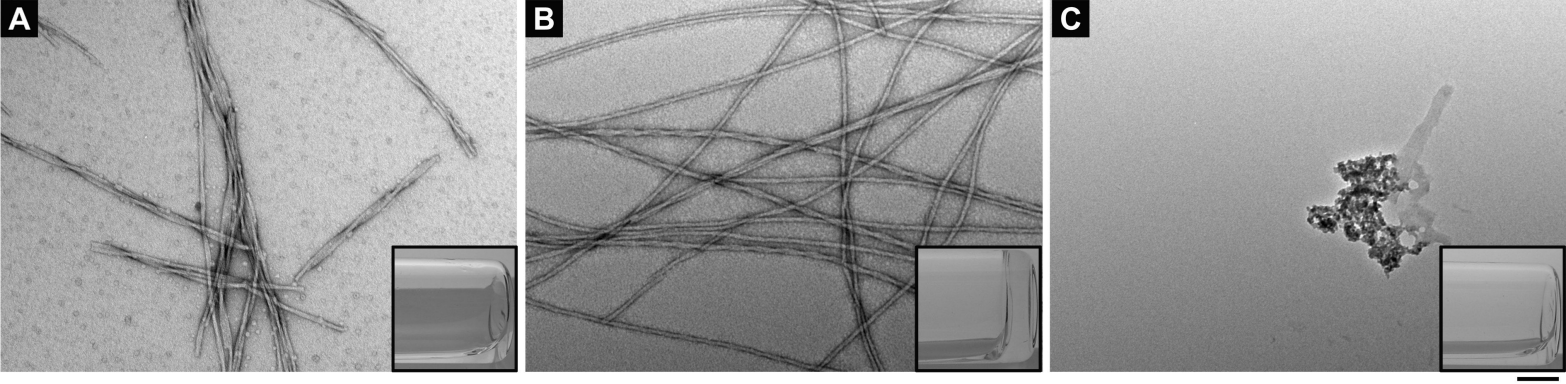
TEM images of (A) solution of **1** + **8**; (B) hydrogel of **5** + **8**; (C) solution of **7** + **8**. Each component is at the concentration of 8.3 mM in PBS buffer (pH 6.2). Inserts are the corresponding optical images. Scale bar is 100 nm.

Supramolecular hydrogels [18–24] formed by self-assembly of small molecules in water, as demonstrated previously by us and other researchers, have numerous potential applications, such as encapsulation and delivery of DNA [25] and microRNA [26], delivery of therapeutic agents [27–29], scaffolds for cell culture [30–31] and spinal arthrodesis [32], sensor for detection of hyperuricemia disease [33] and diabetes [34], and matrix for the electrophoresis of acidic native proteins [35]. After obtaining the pure conjugates **1**–**8**, we tested their gelation properties. Conjugates **1**–**8** show excellent solubility in water. When being mixed in PBS, **5** and **8** ([**5**] = [**8**] = 8.3 mM, pH 6.2) self-assemble to form a hydrogel overnight. The hydrogel of **5** + **8** consists of long and flexible nanofibers (with an average width of 9 ± 2 nm), which entangle to form stable networks (Figure 1B). This result is similar to the hydrogelation when mixing two nucleopeptides of the heterodimer [36]. In contrast, the mixture of **1** and **8** remains a solution. As shown in Figure 1A, the TEM of the solution of **1** + **8** reveals helical nanofibers with an average width of 10 ± 2 nm in the solution. This result indicates that the introduction of the glycan at the C-terminus of **5** increases the solubility of the nanofibers. The mixture of **7** and **8** also fails to result in a hydrogel. Moreover, the TEM of the solution of **7** + **8** hardly exhibits any ordered nanostructure (Figure 1C). This result implies that the base pair interactions between thymine and adenine likely play a critical role for the hydrogelation of the mixture of **5** + **8**. In addition, we did gelation tests for **4** + **5** and **4** + **7**. Both mixtures are unable to self-assemble to form hydrogels at the same conditions used for **5** + **8**. These results illustrate that the subtle change in the molecular structures of the conjugates is able to cause drastically different behaviour of self-assembly [37–38].

**Figure 2:**
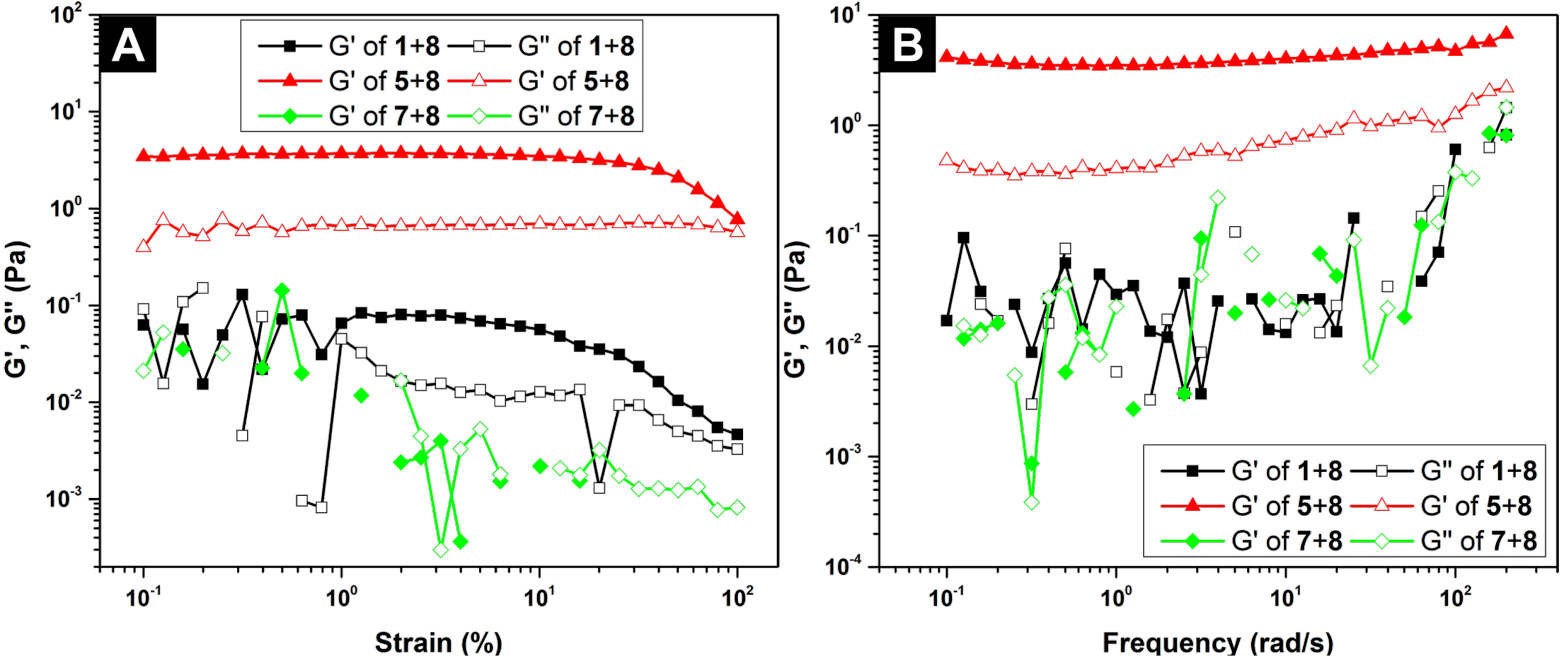
(A) Strain sweep and (B) frequency sweep of the solution of **1** + **8**, the hydrogel of **5** + **8**, and the solution of **7** + **8** at a concentration of 16.6 mM in PBS buffer.

We also investigated the rheological properties of the three mixtures, **1** + **8**, **5** + **8**, and **7** + **8** in PBS buffer. As shown in Figure 2B, storage modulus (G') is higher than loss modulus (G'') for **5** + **8**, confirming that **5** + **8** is a viscoelastic material. Storage moduli (G') overlap with loss moduli (G'') for **1** + **8** and **7** + **8**, agreeing with that **1** + **8** and **7** + **8** behave as liquid-like materials. In addition, the maximum storage for **5** + **8** is 3.7 Pa (Figure 2A), indicating that **5** + **8** is a weak hydrogel. When the strain is between 0.8–100%, the storage modulus of **1** + **8** is slightly higher than that of loss modulus (Figure 2A), which is likely due to the existence of nanofibers in the solution of **1** + **8** (Figure 1A).

### Biostability

**Figure 3:**
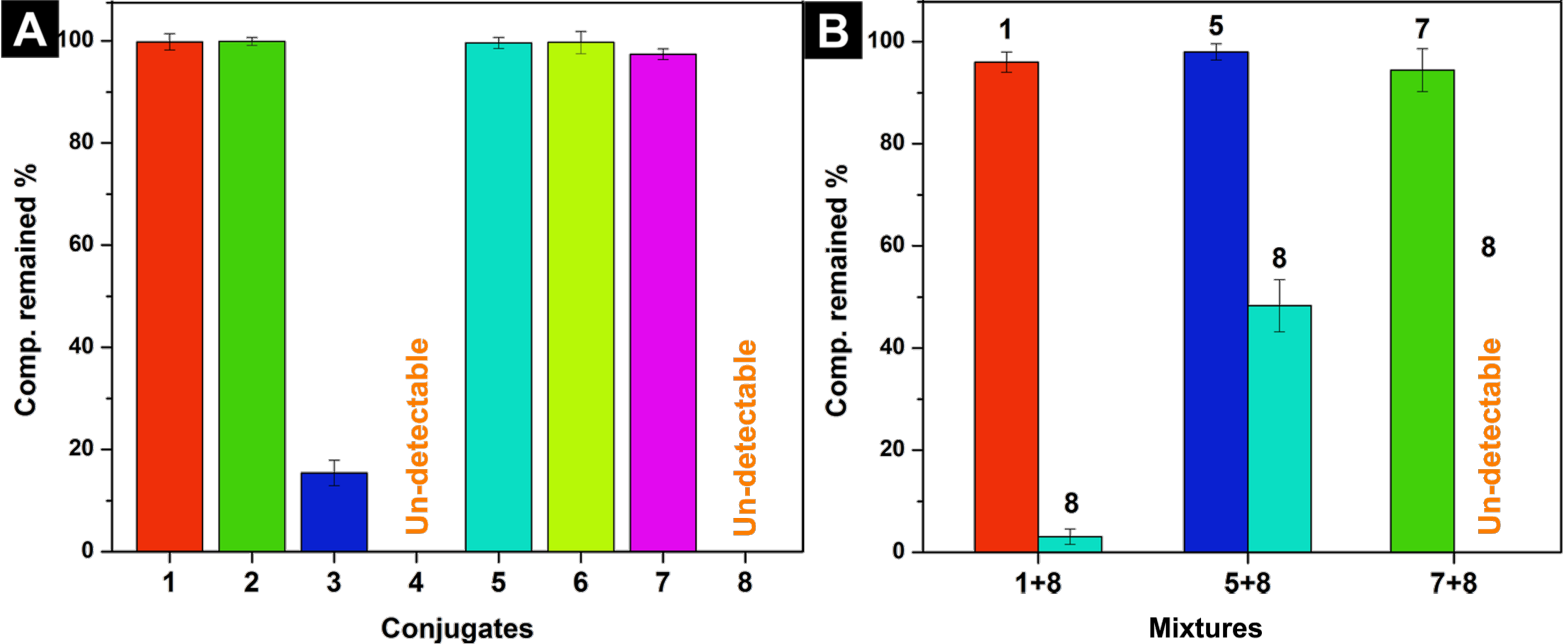
Compounds remained after incubating with proteinase K (3.2 U/mL) for 24 h at 37 °C. (A) Each conjugate **1**–**8** is at the concentration of 0.2 mg/mL in HEPES buffer (pH 7.4). (B) Mixture of **1** + **8**, **5** + **8**, or **7** + **8** is in PBS buffer ([**1**] = [**5**] = [**8**] = 8.3 mM, pH 6.2).

The existence of proteolytic enzymes [39] in organism limits the applications of peptide-based biomaterials [40] in vivo. To evaluate the biostability of the conjugates **1**–**8**, we incubated them with proteinase K (a powerful protease) in HEPES buffer at 37 °C for 24 h. As shown in Figure 3A, conjugates **1**, **2**, **5**, **6**, and **7**, containing peptidic epitopes TTPV or ETPV, almost 100% remained after incubating with proteinase K for 24 h. When we changed the peptide sequence to EPTV, only 15% of **3** left. Conjugates **4** and **8**, containing peptidic epitopes LGFNI, are undetectable after incubating with the proteinase K. The biostability of these conjugates are relevant to their epitopes, since we found that the peptidic epitopes have the same biostability [36]. These results indicate that stable natural peptidic epitopes in the conjugates should be able to improve the biostability of the conjugates. In addition, as shown in Figure 3B, we found that the hydrogel mixture of **5** + **8** promotes the biostability of **8** (about 50% remained at 24 h). The mixture of **7** + **8**, being incubated with proteinase K at the same condition as the test of **5** + **8**, failed to increase the biostability of **8**. The concentration of **8** is slightly increased in the treatment of the mixture of **1** + **8** (about 3% left) with proteinase K, comparing to the case of **8** incubated with proteinase K at 24 h. This result is consistent with TEM investigations showing that there are weak interactions between **1** and **8**.

### Cell compatibility

**Figure 4:**
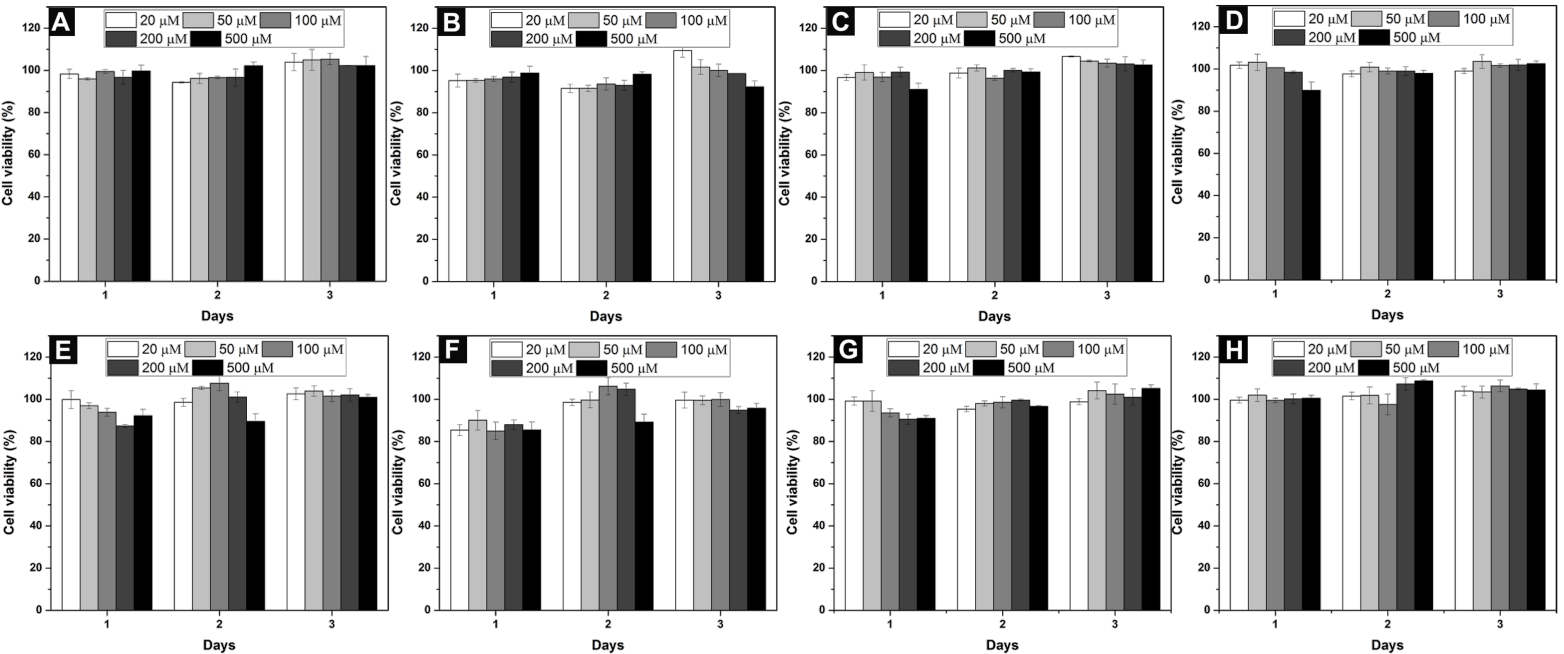
Cell viability of HeLa cells incubated with (A) **1**, (B) **2**, (C) **3**, (D) **4**, (E) **5**, (F) **6**, (G) **7**, (H) **8** at different concentrations for 3 days.

**Figure 5:**
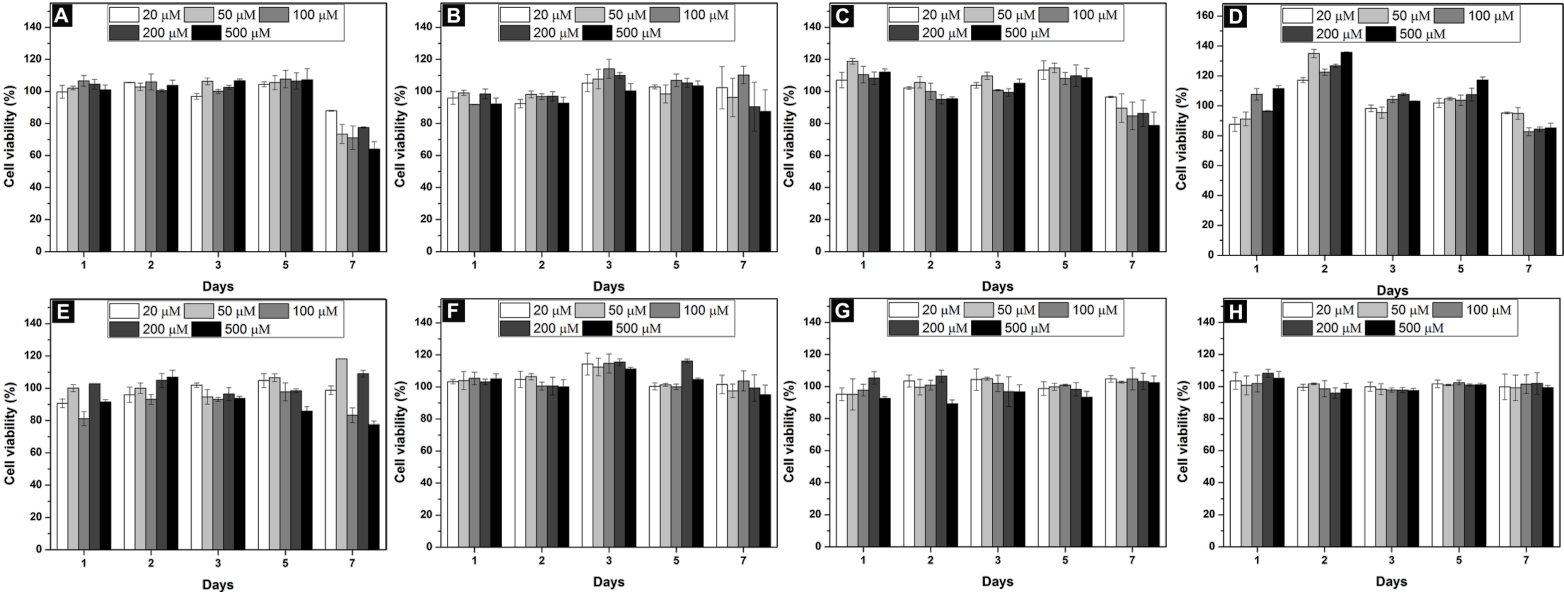
Cell viability of PC12 cells incubated with (A) **1**, (B) **2**, (C) **3**, (D) **4**, (E) **5**, (F) **6**, (G) **7**, (H) **8** at different concentrations for 7 days.

**Figure 6:**
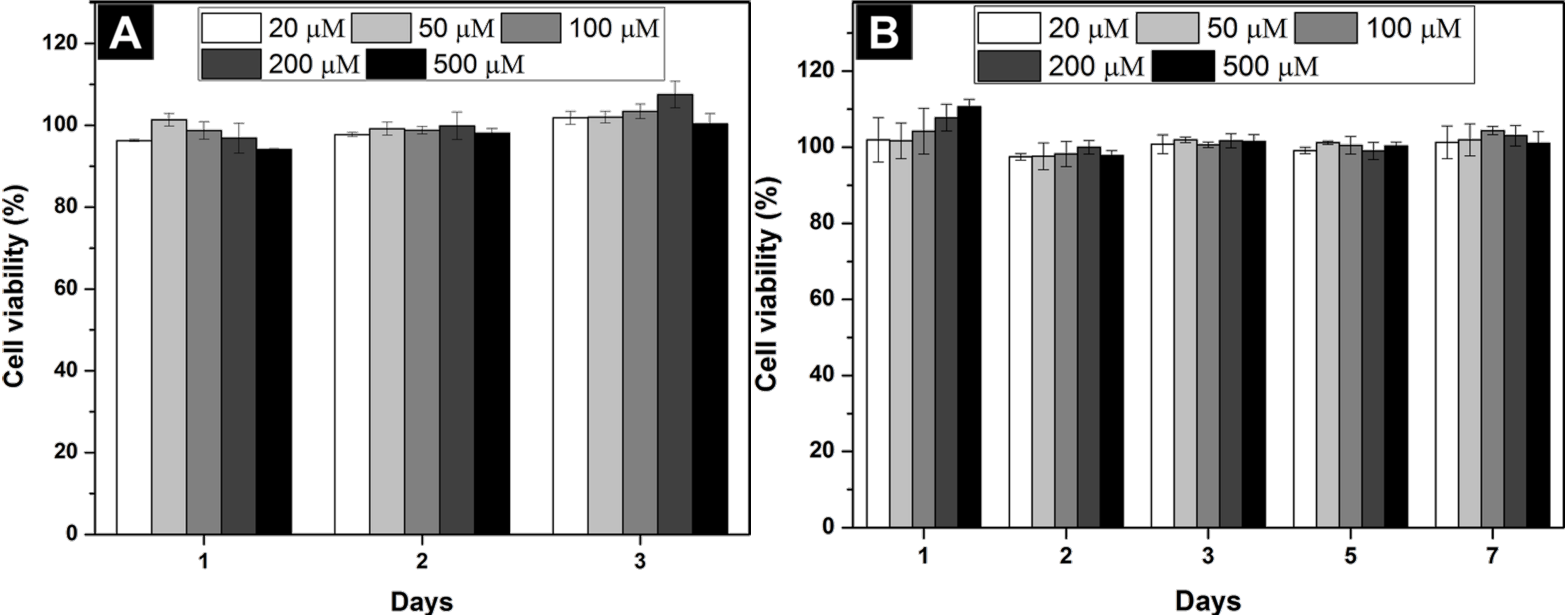
Cell viability of (A) HeLa and (B) PC12 cells incubated with **5** + **8** at different concentrations.

Cell compatibility is one of the major considerations for biomaterials [41–42]. To assess the cell compatibility of the synthesized conjugates, we incubated HeLa and PC12 cells with **1**–**8** at the concentration range of 20–500 μM. As shown in Figure 4, with (**1**–**4**, Figure 4A–D) or without (nucleopeptide, **5**–**8**, Figure 4E–H) glucosamine, conjugates **1**–**8** are innocuous to HeLa cells for treatment of 3 days. Because of a longer doubling time of PC12 than HeLa cells [43–44], we incubated PC12 cells for 7 days. Conjugate **1**–**8** showed little toxicity to PC12 cells (Figure 5). The mixture of **5** + **8**, which has the highest self-assembly ability, also hardly inhibits the proliferation of HeLa and PC12 cells (Figure 6). These results reveal that these conjugates, though having different ability of self-assembly, are cell compatible [2,4,6,9,36]. The cell compatibility of these molecules and the two component hydrogel **5** + **8** promises them to serve as biomaterials.

## Conclusion

In conclusion, we designed eight conjugates by modifying two endogenous binding peptide motifs with nucleobase and glucosamine (or not) and investigated their gelation properties, biostability, and cytotoxicity. Particularly, the mixture of **5** and **8** affords a stable hydrogel, which increases the biostability of **8**. Meanwhile, **5**, **8**, and their mixture show excellent cell compatibility, which is a basic requirement for multi-application in vivo (e.g., wound healing [45]). This work provides a new approach to develop biocompatible soft materials.

## Experimental

### Materials

Starting materials and reagents were purchased from GL Biochem (Shanghai) Ltd. and Fisher Scientific without further purification unless otherwise noted. Proteinase K was purchased from Sigma (>800 unit/mL). The HeLa cell line (CCL-2) and the PC12 (CRL-1721.1) cell line were purchased from the American Type Culture Collection. All of the media were purchased from Invitrogen.

### Instruments

Conjugates were purified with a Water Delta600 HPLC system, equipped with an XTerra C18 RP column and an in-line diode array UV detector. ^1^H NMR spectra were obtained on a Varian Unity Inova 400 spectrometer. LC–MS spectra were performed on Waters Acquity Ultra Performance LC with Waters MICROMASS detector. TEM images were taken on a Morgagni 268 transmission electron microscope. Rheological data were measured on a TA ARES G2 rheometer with 25 mm cone plate. MTT assay for cell toxicity test were measured on a DTX880 Multimode Detector.

### Synthesis

The synthesis procedures for conjugates **1**–**8** are demonstrated in the main text synthesis part.

**1**: ^1^H NMR (400 MHz, D_2_O) δ 7.89 (d, *J* = 8.6 Hz, 1H), 7.83 (d, *J* = 8.6 Hz, 1H), 7.73 (d, *J* = 7.6 Hz, 1H), 7.65–7.59 (m, 1H), 7.44 (s, 1H), 5.20 (d, *J* = 3.5 Hz, 1H), 4.68–4.59 (m, 4H), 4.53–4.41 (m, 3H), 4.26–4.15 (m, 3H), 3.92–3.69 (m, 6H), 3.51–3.45 (m, 1H), 2.35–2.27 (m, 1H), 2.16–1.91 (m, 4H), 1.89 (s, 3H), 1.26–1.21 (m, 6H), 1.00–0.88 (m, 6H); LC–MS (ESI) *m*/*z*: [M − 1]^−^ calcd for C_31_H_49_N_7_O_14_, 743.77; found, 742.41.

**2**: ^1^H NMR (400 MHz, D_2_O) δ 7.43 (s, 1H), 5.25–5.14 (m, 1H), 4.62–4.46 (m, 5H), 4.17–4.08 (m, 3H), 3.94–3.39 (m, 9H), 2.51 (t, *J* = 7.4 Hz, 2H), 2.34–1.92 (m, 7H), 1.90 (s, 3H), 1.26 (d, *J* = 6.3 Hz, 3H), 1.04–0.98 (m, 6H); LC–MS (ESI) *m*/*z*: [M − 1]^−^ calcd for C_32_H_49_N_7_O_15_, 771.78; found, 770.56.

**3**: ^1^H NMR (400 MHz, D_2_O) δ 8.17 (d, *J* = 8.4 Hz, 1H), 7.42 (s, 1H), 5.19 (d, *J* = 3.4 Hz, 1H), 4.65–4.46 (m, 4H), 4.34 (d, *J* = 5.4 Hz, 1H), 4.29–4.08 (m, 3H), 3.97–3.41 (m, 9H), 2.58–2.54 (m, 2H), 2.42–2.26 (m, 1H), 2.22–1.94 (m, 6H), 1.90 (s, 3H), 1.32–1.16 (m, 3H), 1.01–0.91 (m, 6H); LC–MS (ESI) *m*/*z*: [M − 1]^−^ calcd for C_32_H_49_N_7_O_15_, 771.78; found, 770.56.

**4**: ^1^H NMR (400 MHz, DMSO-*d*_6_) δ 11.23 (s, 1H), 8.49 (d, *J* = 7.8 Hz, 1H), 8.39–8.28 (m, 1H), 8.17–8.03 (m, 2H), 7.77 (d, *J* = 7.5 Hz, 1H), 7.67 (d, *J* = 7.2 Hz, 1H), 7.56–7.48 (m, 1H), 7.38 (s, 2H), 7.26–7.16 (m, 5H), 6.99–6.90 (m, 1H), 4.93 (s, 1H), 4.70–4.18 (m, 6H), 3.97–3.44 (m, 12H), 3.17–2.91 (m, 2H), 2.77–2.65 (m, 1H), 2.42, (m, 1H), 1.82–1.75 (m, 1H), 1.72 (s, 3H), 1.66–1.52 (m, 1H), 1.47–1.39 (m, 3H), 1.35–1.28 (m, 1H), 1.08–0.99 (m, 1H), 0.90–0.69 (m, 12H); LC–MS (ESI) *m*/*z*: [M − 1]^−^ calcd for C_40_H_59_N_9_O_14_, 889.96; found, 888.58.

**5**: ^1^H NMR (400 MHz, D_2_O) δ 7.29 (s, 1H), 4.49–4.46 (m, 3H), 4.37–4.23 (m, 2H), 4.14–3.91 (m, 3H), 3.81–3.69 (m, 1H), 3.63–3.51 (m, 1H), 2.20–1.97 (m, 2H), 1.95–1.76 (m, 3H), 1.74, (s, 3H), 1.12–1.02 (m, 6H), 0.90–0.76 (m, 6H); LC–MS (ESI) *m*/*z*: [M − 1]^−^ calcd for C_25_H_38_N_6_O_10_, 582.61; found, 581.19.

**6**: ^1^H NMR (400 MHz, D_2_O) δ 7.43 (s, 1H), 4.68–4.41 (m, 4H), 4.28–4.07 (m, 2H), 3.93–3.84 (m, 1H), 3.81–3.68 (m, 1H), 2.51 (t, *J* = 7.1 Hz, 2H), 2.34–1.93 (m, 7H), 1.90 (s, 3H), 1.26 (d, *J* = 6.4 Hz, 3H), 1.03–0.98 (m, 6H); LC–MS (ESI) *m*/*z*: [M − 1]^−^ calcd for C_26_H_38_N_6_O_11_, 610.62; found, 609.34.

**7**: ^1^H NMR (400 MHz, D_2_O) δ 8.37 (s, 1H), 8.26 (s, 1H), 7.14–7.00 (m, 3H), 6.79–6.71 (m, 2H), 5.10–4.96 (m, 2H), 4.57 (t, *J* = 8.0 Hz, 2H), 4.45–4.38 (m, 1H), 4.26–4.18 (m, 1H), 3.56 (t, *J* = 6.2 Hz, 2H), 3.06–2.70 (m, 4H), 2.27–2.19 (m, 2H), 2.02–1.94 (m, 3H), 1.17–1.13 (m, 6H), 1.02–0.95 (m, 6H); LC–MS (ESI) *m*/*z*: [M − 1]^−^ calcd for C_25_H_37_N_9_O_8_, 591.63; found, 590.35.

**8**: ^1^H NMR (400 MHz, DMSO-*d*_6_) δ 8.60 (d, *J* = 7.8 Hz, 1H), 8.41 (d, *J* = 7.6 Hz, 1H), 8.32 (d, *J* = 15.4 Hz, 2H), 8.24 (d, *J* = 5.8 Hz, 1H), 8.05 (d, *J* = 8.3 Hz, 1H), 7.77 (d, *J* = 8.5 Hz, 1H), 7.37 (s, 1H), 7.26–7.13 (m, 5H), 6.94 (s, 1H), 4.98 (s, 2H), 4.69–4.58 (m, 2H), 4.55–4.49 (m, 2H), 4.34–4.28 (m, 1H), 4.22–4.14 (m, 1H), 3.72 (dd, *J* = 16.4, 5.9 Hz, 1H), 3.55 (dd, *J* = 16.3, 5.3 Hz, 1H), 3.04–2.95 (m, 1H), 2.73–2.62 (m, 1H), 2.59–2.53 (m, 1H), 2.43–2.37 (m, 1H), 1.82–1.75 (m, 1H), 1.66–1.55 (m, 1H), 1.50–1.34 (m, 3H), 1.21–1.10 (m, 1H), 0.93–0.78 (m, 12H); LC–MS (ESI) *m*/*z*: [M − 1]^−^ C_34_H_47_N_11_O_8_, 737.82; found, 736.67.
